# High-yielding ^18^F radiosynthesis of a novel oxytocin receptor tracer, a probe for nose-to-brain oxytocin uptake *in vivo*†
†Electronic supplementary information (ESI) available. See DOI: 10.1039/c8cc01400k


**DOI:** 10.1039/c8cc01400k

**Published:** 2018-07-05

**Authors:** Rhiannon Beard, Nisha Singh, Christophe Grundschober, Antony D. Gee, Edward W. Tate

**Affiliations:** a Department of Chemistry , Imperial College London , Exhibition Road , London , SW7 2AZ , UK . Email: rhiannon.beard@gmail.com ; Email: e.tate@imperial.ac.uk; b Division of Imaging Sciences , King's College London , 4th Floor , Lambeth Wing , St Thomas’ Hospital , London , SE1 7EH , UK . Email: antony.gee@kcl.ac.uk; c Centre for Neuroimaging Sciences , IoPPN , KCL , De Crespigny Park , SE5 8AF , London , UK; d Roche Pharma Research and Early Development , Discovery Neuroscience , Roche Innovation Center Basel , F. Hoffmann-La Roche Ltd , Grenzacherstrasse 124 , 4070 Basel , Switzerland

## Abstract

A novel Al^18^F labelled peptide tracer for PET imaging of oxytocin receptor has been accessed through a high radiochemical yield approach. This tracer showed comparable affinity and higher selectivity and stability compared to oxytocin, and was used to demonstrate direct nose-to-brain uptake following intranasal administration, a common yet controversial delivery route for oxytocin-based therapeutics.

## Abstract 2

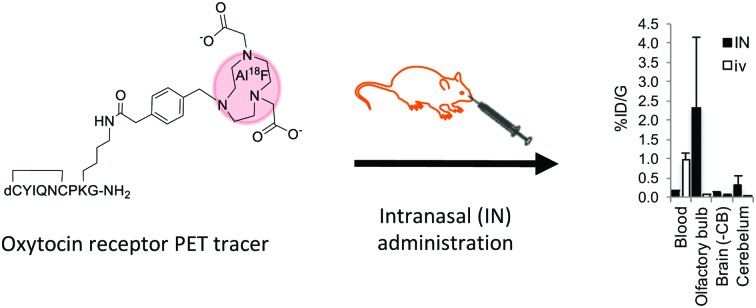
Probing the mechanism of intranasal oxytocin brain uptake through generation and validation of a novel peptide PET tracer.

## 

The oxytocin (OT) system comprises the peptide hormone OT and the transmembrane G-protein coupled oxytocin receptor (OTR), which is expressed in organs including the uterus, heart, kidney and brain. OT, the endogenous ligand of OTR, is produced within the hypothalamus and stored in the posterior pituitary gland until release after stimulation such as hugging, breast-feeding and sexual intercourse.^1^ Consequently, OT has been termed the “love hormone”, as it facilitates reproduction at all levels. More recently, the putative role of OT in modulation of complex neuropsychiatric disorders including autism spectrum disorders (ASD) and schizophrenia where it has undergone investigation as a therapeutic, has been the subject of considerable controversy.^2^–^4^ In one study, mice with a mutation in Cntnap2, which in humans leads to autistic spectrum disorders, experienced social deficits and lower levels of brain OT. Interestingly, daily treatment with OT reversed behavioural deficits.^3^ Further, studies in humans have shown positive effects for treatment of core symptoms of ASD when OT is administered intravenously (i.v.).^4^ However, the blood–brain barrier severely restricts circulating OT from crossing into the central nervous system, limiting translation of basic OT research into effective and much needed treatment solutions for behavioural disorders.

Intranasal (i.n.) administration offers the potential for non-invasive central nervous system delivery in a direct nose-to-brain fashion.^5^ Multiple reports suggest acute OT administration affects social behaviours when given as a nasal spray in humans.^6^,^7^ However, clinical trials that study the prolonged administration of OT have failed to demonstrate any clear therapeutic benefit by various clinical endpoints.^8^,^9^ The mechanism of putative OT nose-to-brain delivery is poorly understood, but is conjectured to occur either directly through olfactory and nerve fibre routes, or indirectly *via* absorption into blood capillaries.^5^,^10^ Currently no studies have quantified brain uptake or distribution of OT after i.n. administration, since measurements of this kind are typically invasive. We considered that a functional *in vivo* OTR positron emission tomography (PET) tracer could provide a useful biological tool for mapping OTR expression and enable a sensitive, non-invasive route to quantify brain uptake and distribution *via* i.n. administration of OT-based therapeutics.

^18^F (*T*_1/2_ 100 min, β+ 0.635 MeV 97%) is the most frequently used isotope for PET owing to its almost ideal imaging properties, including low positron energy, an extended half-life and lack of side emissions. Fluoride labelling strategies most widely applied for PET tracer synthesis of biomolecules use nucleophilic or electrophilic chemistries to attach ^18^F to carbon as a “prosthetic group”, with these reagents requiring prior synthesis. In contrast, direct radiolabelling significantly simplifies and shortens radiosynthesis by late-stage incorporation of ^18^F,^11^ for example using NODA-derived chelation cages to irreversibly bind Al^18^F in high yield.^12^,^13^ The core architecture of our tracer was design to maintain selective receptor binding while allowing direct [^18^F]AlF labelling (Fig. 1A–D). Consequently the tracer core was based on dLVT, an OT analogue shown to accommodate large groups on position 8 whilst retaining affinity and receptor selectivity (Fig. 1B).^14^,^15^ The dLVT peptide architecture was produced using automated Fmoc-synthesis incorporating a protected deaminated cysteine residue (dC) in the final position (ESI,† Scheme S1), reported to enhance OT stability *in vivo*.^16^,^17^ Orthogonal deprotection of the lysine 1-(4,4-dimethyl-2,6-dioxocyclohex-1-ylidene)ethyl (ivdde) allowed NODA conjugation on solid support *via* a methylphenylacetic acid (MPAA) linker. Following conjugation, full deprotection and cleavage from the resin afforded crude peptide which was purified by RP-LCMS. Cyclisation was performed through DMSO-mediated oxidation, affording final product in 22% overall yield. To enable molar activity and *K*_i_ determination, cold (non-radioactive) AlF-NODA-dLVT was also synthesised, alongside a library of OT peptide analogues (Table 1) to determine a structure–activity relationship (SAR) for OTR binding. Full sequences for these peptides can be found in ESI,† Table S1.

**Fig. 1:**
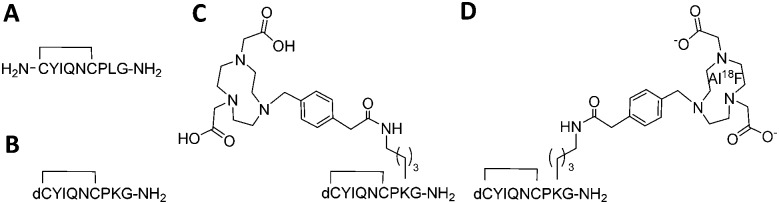
Structure of OT (A), dLVT (B), OTR tracer precursor NODA-dLVT (C), and ^18^F radiolabelled tracer (D). Peptides contain a cyclic configuration due to the disulphide bond between positions 1 and 6. Key: dC = deaminated cysteine.

**Table 1:** *K*_i_ values (nM) of OT and peptide analogues for human OTR and vasopressin receptors

Peptide	OTR	V1_a_	V1_b_	V_2_
OT_c_	1.2 ± 0.3	20 ± 3	>6000	>6000
dOT_c_	1.6 ± 1.3	388 ± 157	nd	>6000
dOT_oc_	1.2 ± 0.9	362 ± 124	nd	>6000
dLVT_c_	3.4 ± 1.0	24 ± 5	nd	4844 ± 1670
dLVT_oc_	6.3 ± 3.6	31 ± 9	nd	5819 ± 1704
AlF-NODA-dLVT_c_	2.6 ± 1.7	157 ± 26	>6000	>6000
AlF-NODA-dLVT_oc_	3.3 ± 1.5	821 ± 217	nd	>6000

Development of a highly specific ligand for OTR is challenging since endogenous OT and arginine vasopressin (AVP) peptides and their corresponding receptors share a related structure. OT analogues thus have the potential to bind to multiple members of the OT/AVP receptor family comprising the OTR and three AVP receptor subtypes, and therefore we determined AlF-NODA-dLVT's binding profile across these potential targets.

OT analogues were screened in scintillation proximity assays that displace [^3^H]OT or [^3^H]AVP from the corresponding receptors. All peptides screened, including in a reduced state, preferentially bound human OTR with nanomolar affinity (Table 1). In line with previous studies, conjugation of a large group to position 8 afforded analogues with increased selectivity and improved affinity towards OTR^15^ (approximately 60-fold for AlF-NODA-dLVT, and 7-fold for dLVT; Table 1 and ESI,† Fig. S1). AlF-NODA-dLVT also demonstrated enhanced selectivity (approximately 60-fold) towards OTR compared to natural OT (approximately 16-fold). Remarkably, selectivity was improved when the tracer was in a reduced state (>240-fold), with similar affinity towards the OTR.

Encouraged by these results, a radiofluorination method for the preparation of [^18^F]AlF-NODA-dLVT was investigated. In the course of developing this reaction we identified a substantial loss of activity during concentration of the radionuclide. Following the reported procedure, [^18^F]fluoride is trapped on an anion exchange resin preconditioned with sodium acetate buffer, and ^18^F is concentrated into 100 μL fractions through elution with saline, at an optimal pH for subsequent peptide labelling.^13^ Under these conditions, 0.9% saline produced a broad Na^18^F elution profile (Fig. 2A).

**Fig. 2:**
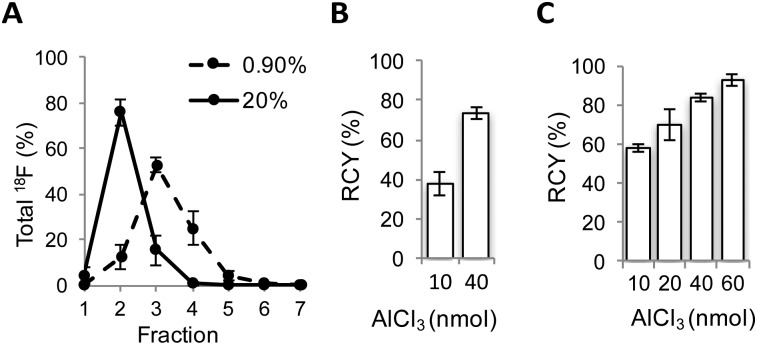
Optimisation of ^18^F radiolabelling of NODA-dLVT. (A) Elution profile of [^18^F]NaF with 0.9% (dashed) or 20% (solid) saline from anion exchange resin in 100 μL fractions, mean of 5 samples for each condition. Radiochemical conversion (RCC) for [^18^F]AlF labelling of NODA-dLVT (72 nmol) using 0.9% (B) or 20% (C) saline with different molar equivalents of aluminium chloride at 105 °C for 15 min. Values represent mean of 3 samples.

Consequently only 53 ± 3% Na^18^F was isolated in the highest activity fraction. To overcome this loss, we narrowed the elution profile using higher saline concentration, and found that 20% saline increased isolation yields, allowing 76 ± 5% of total ^18^F activity to be eluted in the highest activity fraction (Fig. 2A). These values are improved over previous reports that compensate for this loss by eluting Na^18^F in larger volumes from 2-fold^13^ to 5-fold.^18^ Dilution limits the activity used for radiolabelling since peptide concentration heavily influences RCY, resulting in overall RCY of 8.2% (from end of bombardment (EOB)), despite high radionuclide incorporation and purification (86%).^18^ In further optimisation experiments, radiolabelling was performed following ^18^F elution with the highest activity fraction eluted with either 0.9% or 20% saline, and with various molar equivalents of AlCl_3_ compared to peptide precursor. 20% saline consistently improved radiochemical conversion (RCC) compared to 0.9% saline under analogous conditions (Fig. 2B and C and ESI,† Fig. S2). For example, with 10 nmol aluminium chloride a 1.5-fold increase in RCC of [^18^F]AlF-NODA-dLVT was observed with 20% saline (Fig. 2C). These yields are similar to those obtained when organic co-solvent is added to the mixture,^19^,^20^ and may offer an alternative method when DMSO or MeOH is not suitable for the precursor, for example for peptides sensitive to oxidation and for larger biomolecules. This marked improvement was translatable to an unrelated model peptide, NODA-GFAG, although the improvement in RCC was less pronounced (ESI,† Fig. S3). Increased molar ratios of aluminium chloride to peptide gave higher conversion for [^18^F]AlF-NODA-dLVT, however, we found that lower concentrations of aluminium chloride were preferred to yield higher molar radioactivity in the isolated tracer; 60 nmol of aluminium chloride (1 : 1.2 aluminium chloride : peptide molar ratio) afforded [^18^F]AlF-NODA-dLVT in RCC of 90 ± 3% with a molar radioactivity of 39 ± 12 GBq μmol^–1^, whereas 20 nmol (*i.e.* 1 : 3.6 aluminium chloride : peptide) afforded the tracer in 71 ± 5% RCC, but with a molar activity of 66 ± 8% GBq μmol^–1^. Using optimised conditions, [^18^F]AlF-NODA-dLVT was synthesised reproducibly and formulated within 70 min, with isolated RCY of 36% (decay corrected, from EOB), radiochemical purity (RCP) ≥ 95% and molar activity ≥ 58 GBq μmol^–1^ (ESI,† Fig. S4). Notably, the RCY reported here is significantly improved from previous reports using a similar labelling approach, which range from 8.2–24% (from EOB).^18^,^20^–^22^

To justify use of [Al^18^F]NODA-dLVT *in vivo*, tracer lipophilicity and specific binding were determined through a standard shake flask assay and rat brain autoradiography, respectively. A log *D*_7.4_ of –0.54 ± 0.1 was established for [Al^18^F]NODA-dLVT indicating a hydrophilic profile and potential for low non-specific binding. To investigate the feasibility of [Al^18^F]NODA-dLVT for nervous system imaging, tracer was incubated at a concentration of 3 nM, either alone or in the presence of excess OT (10 μM) with brain tissue cut in the sagittal plane to determine total *vs.* non-specific binding (Fig. 3). This enabled calculation of % specific binding of [Al^18^F]NODA-dLVT to regions of brain tissue (ESI,† Section 4.2). [^18^F]AlF-NODA-dLVT specifically bound regions known to express appreciable levels of OTR (Fig. 3),^23^–^26^ including hippocampus (CA1: 53 ± 4%; *subiculum*, S: 60 ± 1%), central amygdala nucleus (ac: 56 ± 1%), olfactory systems (olfactory tubercle, Tu: 53 ± 5%; anterior olfactory nucleus, AO: 38 ± 2%), acumens nuclei (Acb: 43 ± 4%), and cortical regions (visual cortex, V1: 61 ± 4%; motor cortex, MC: 42 ± 4%).

**Fig. 3:**
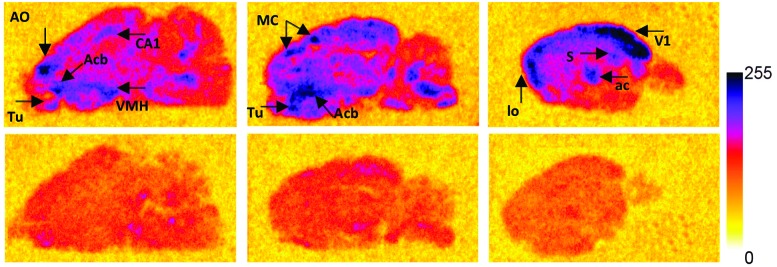
Specific binding profile of [^18^F]AlF-NODA-dLVT. Radiograms of total (top; 3 nM [^18^F]AlF-NODA-dLVT) and nonspecific binding (bottom; 3 nM [^18^F]AlF-NODA-dLVT and 10 μM OT) of [^18^F]AlF-NODA-dLVT to rat brain slices in the sagittal pane. Areas with high specific binding are indicated on the figure with arrow heads. Key: AO = anterior olfactory nucleus; CA1 = field CA1 of the hippocampus; Acb = accumbens nuclei; Tu = olfactory tubercle; VMH = ventromedial hypothalamic nuclei; MC = motor cortex; V1 = primary visual cortex; S = *subiculum*; ac = central amygdala nucleus; lo = lateral olfactory tract.

To investigate the plausibility of i.n. administration for OT-like therapies, given the similar structure and hydrophilic profile between AlF-NODA-dLVT and OT, *in vivo* tissue distribution of [^18^F]AlF-NODA-dLVT was determined following i.n. or i.v. administration. Since OT is rapidly degraded following i.v. injection (plasma half-life approximately 3 min),^16^ the systemic plasma half-life of AlF-NODA-dLVT was first determined after i.v. bolus (ESI,† Fig. S5). AlF-NODA-dLVT had an approximately 5-fold increase in half-life compared to OT (14 min *vs.* 3 min), and time-points of 10 min and 30 min were thus selected for i.v. and i.n. administration routes, respectively. Here, it is anticipated that both routes show independent kinetic and metabolic profiles and the extended apparent availability of OT following i.n. administration supports a longer timeframe for this study.^27^,^28^ Distribution was determined through gamma counting of radioactivity in isolated tissue. A large portion underwent excretion *via* kidneys (i.v. 4.6 ± 0.4%ID per g; i.n. 1.31 ± 0.7%ID per g) and urine (i.v. 2.5 ± 1.2%ID per g; i.n. 7.7 ± 2.2%ID per g), as expected from the hydrophilic character of the tracer, similar to previous results found with radiolabelled OT given i.v. (Fig. 4A).^29^

**Fig. 4:**
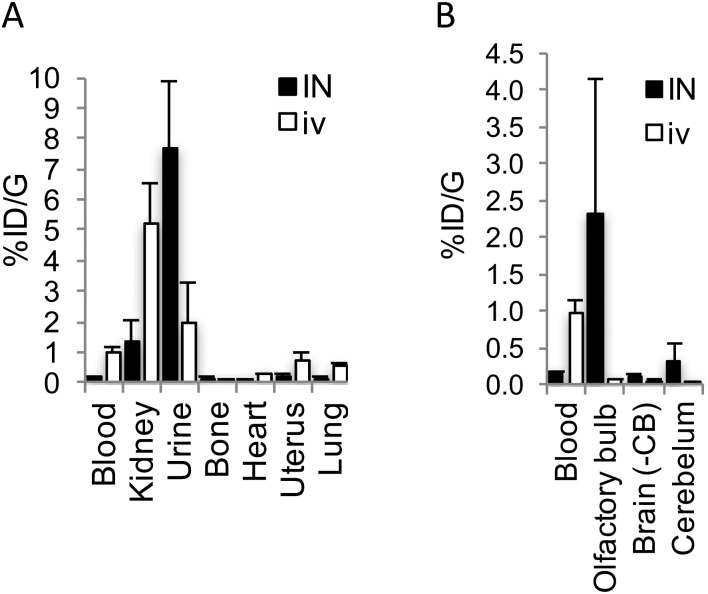
Biodistribution of [^18^F]AlF-NODA-dLVT in rat. (A) Shows biodistribution in major peripheral organs, while (B) shows uptake of [^18^F]AlF-NODA-dLVT in central nervous system regions. Both are shown relative to blood levels following either i.n. (30 min) or i.v. (10 min) administration. Each data point corresponds to mean ± SD, *n* = 3.

[^18^F]AlF-NODA-dLVT uptake in brain parenchyma was increased by i.n. administration, however exposure remained low (i.v. 0.05 ± 0.03%ID per g; i.n. 0.11 ± 0.03%ID per g, *P* = 0.044; Fig. 4B), which was mirrored in nanoPET scans over the course of 1.5 h (ESI,† Fig. S6). Accounting for plasma volume contributions from the 5.2% of the brain assumed to be capillaries, the brain permeable material in this study is approximately 4.7 × 10^–2^%ID per g by i.v. administration.^30^ A significant proportion was detected in the olfactory bulb following i.n. administration compared to the i.v. route (i.n. 2.30 ± 1.8 *cf.* i.v. 0.05 ± 0.02%ID per g, *P* = 0.026), implying nose-to-brain uptake *via* nerve fibres, as opposed to blood-to-brain transfer from indirect diffusion into the bloodstream.^31^,^32^ In addition, a large portion was detected in the cerebellum following i.n. administration in relation to parenchyma uptake (i.n. 0.31 ± 0.26; 2.8-fold *cf.* i.v. 0.05 ± 0.01%ID per g; 1.1-fold).

In summary, we have developed a novel OTR tracer NODA-dLVT radiolabelled with Al^18^F, that binds OTR with nanomolar affinity, and exhibits an enhanced selectivity profile compared to OT in both oxidised and reduced states. During the radiosynthesis, we incorporated an improved methodology for aqueous ^18^F fluoride recovery which significantly enhances RCY, and is likely to be widely applicable to other radiotracers. [^18^F]AlF-NODA-dLVT administered by i.n. and i.v. routes demonstrated low brain parenchyma uptake. Yet, i.n. administration gave a significant signal in the olfactory bulbs suggesting “direct nose-to-brain” uptake. However, it is important to note that the tracer was unable to penetrate deeper regions of brain tissue for specific binding during the timescale studied. Consequently, our findings suggest that the i.n. route is not significantly more efficient than i.v. for OT-based tracers. While i.n. administration lead to modest parenchyma delivery, this increase may be relevant for changes in behavioural and neural responses, as suggested in other studies. In addition, the presence of radioactivity in the cerebellum may suggest peptide degradation following absorption, and improvement in the stability and selectivity of OT agonists may prove beneficial for clinical efficacy.

The expert technical assistance of Andy Stucki, Muriel Schmitt and Gabrielle Py for the radioligand binding is gratefully acknowledged. This work was supported by a grant to the Institute of Chemical Biology from the EPSRC (EP/F500416/1), the Medical Research Council (MR/K022733/1), the Centre of Excellence in Medical Engineering by the Wellcome Trust and EPSRC (WT 088641/Z/09/Z), the Innovative Medicines Initiative Joint Undertaking under grant agreement (No. 115300), resources of which are composed of financial contribution from the European Union's Seventh Framework Programme (FP7/2007-2013) and EFPIA companies' in kind contribution, and an Independent Researcher Award (King's College London).

## Conflicts of interest

There are no conflicts to declare.

## Supplementary Material

Supplementary information
