# Supplementary material for: High-yielding 18F radiosynthesis of a novel oxytocin receptor tracer, a probe for nose-to-brain oxytocin uptake in vivo
Source: Chem Commun (Camb). 2018 Jul 5;54(58):8120–3. doi: 10.1039/c8cc01400k (PMC6049614; doi:10.1039/c8cc01400k)
Supplement: Supplementary file 1 [file CC-054-C8CC01400K-s001.pdf]

Electronic Supplementary Material (ESI) for ChemComm.
This journal is © The Royal Society of Chemistry 2018

High-yielding 18F radiosynthesis of a novel oxytocin receptor tracer, a probe for nose-to-brain oxytocin uptake *in vivo*

Rhiannon Beard[1]; Nisha Singh[2,3]; Christophe Grundschober[4]; Antony D. Gee[2] and Edward W. Tate[1]

[a,1]Department of Chemistry, Imperial College London, Exhibition Road, London, SW7 2AZ, UK; [2]Division of Imaging Sciences, King's College London, 4th Floor, Lambeth Wing, St Thomas' Hospital, SE1, 7EH, London, UK; [3]Centre for Neuroimaging Sciences, IoPPN, KCL, De Crespigny Park, London, SE5 8AF, UK; and [4] Roche Pharma Research and Early Development, Discovery Neuroscience, Roche Innovation Center Basel, F. Hoffmann-La Roche Ltd, Grenzacherstrasse 124, 4070 Basel, Switzerland

SUPPORTING DATA

1 General Considerations

All RCYs reported are decay corrected to the time of ^{18}F delivery. The protocols for performing receptor radioligand binding and potency studies on transfected cells to determine affinity constants have been reported elsewhere.[1]

All general laboratory chemicals were obtained from chemical suppliers (Novabiochem UK or Sigma-Aldrich Chemical Co.). NO2AtBu was purchased from CheMatech, and QMA cartridges (WAT023525) were from Waters.

Purification of synthesized crude peptides and analysis was performed on a Waters LC-MS system (Waters 2767 autosampler for samples injection and collection; Waters 515 HPLC pump to deliver the mobile phase to the source; Waters 3100 mass spectrometer with ESI; and, Waters 2998 Photodiode Array (detection at 200–600 nm)), equipped with an X Bridge C18 reverse-phase column (4.6 mm D x 100 mm, 5 μm) for analytical and 19 mm D × 100 mm L for preparative. Radiochemical analysis and purification were performed on an Agilent 1200 Series HPLC system (including degasser, Quat pump, VWD and manual injector), and was coupled to a Raytest Gabi Star detector with a sodium iodide probe (B-FC-3200). Aliquots (20 – 100 μl) of radiolabeled sample was injected onto Agilent Extend-C18 (4.6 x 150 mm, 5 μm) column for analytical samples, or Agilent Eclipse XDB-C18 (9.4 x 250 mm, 5 μm) column for

semi-prep, using a flow rate of 1 mL/min or 4 mL/min for the respective columns.

1.1 Purification and analytical methods

Water with 0.1% formic acid or 0.1% TFA was used as the secondary solvent in the reverse phase liquid chromatography mass spectrometry (RP-LCMS) and reverse phase high pressure liquid chromatography (RP-HPLC) methods respectively. Retention times (R_t) are reported in Supplementary Table 1.

RP-LCMS: 5% to 98% MeOH in H_2O over 15 min. All solvents were degassed with helium.

All hot analysis and purification was conducted on the specified RP-HPLC system.

RP-HPLC: Analysis: 2% MeCN (with 0.1% formic acid) at 4 min, increasing to 20% at 25 min, which was held for 5 min. The gradient was further increased to 98% over 2 min and returned to the starting gradient to finish at 35 min. Purification: 2% MeCN at 4 min, increasing to 25% at 14 min, then 40% at 27 min and 80% at 29 min, returning to the starting gradient to finish at 35 min.

2 Solid Phase Peptide Synthesis (SPPS) and modifications of OT analogues

Rink-amide resin, used in the synthesis of all OT analogues, was purchased from Novabiochem UK, with a substitution of 0.71 mmol/g. Peptides were synthesised using automated solid-phase peptide synthesis on a ResPep SL apparatus (Intavis, Germany). When applicable (i.e. in the synthesis of deaminated OT (dOT), dLVT, and NODA-dLVT), deaminated cysteine (dC) was coupled under standard conditions at the terminal position of the peptide. The procedure for the synthesis of dC and NODA-MAPP has been reported elsewhere.[2-3] Characterisation data of peptides are reported in Supplementary Table 1.

2.1 SPPS Protocol

Before starting, resin (20 µmol per well) was swelled in DMF for a minimum of 30 minutes.

Within the synthesis a fivefold excess of amino acid over resin reactive groups (100 μmol, 200 μL of 0.5 M solution) was used for each coupling. *In situ* activation and coupling was carried out for 45 min with a mixture of HBTU–HOBt (100 μmol, 200 μL of a 0.5 M solution) and NMM (100 μmol, 200 μL of 0.5 M solution). The peptide was washed with DMF (3 x 1 mL) between each deprotection and coupling step. A typical cycle consisted of Fmoc deprotection, DMF wash, coupling and a further DMF wash. *N*-α-Fmoc protection at the final residue was removed at the end of the synthesis under usual conditions. Following synthesis, the peptidyl resin was removed from the synthesizer, washed several times (3 x 2 mL DMF, 3 x 2 mL DCM, 3 x 2 mL methanol, 3 x 2 mL diethylether) and dried in a desiccator over silica gel, unless otherwise stated. For deprotection and cleavage from the resin, a standard 'deprotection mixture' of TFA–H_2O–DTT–TIS (94 : 2.5 : 2.5 : 1) was used in all reactions (1.5 ml of mixture to 20 μmol of dry peptide bound resin), unless otherwise stated. After cleavage, crude mixtures were precipitated using ice cold TBME, centrifuged at 4000 rpm for 10 minutes at 4 ^{o}C, supernatants were discarded and the remaining peptides were washed with a fresh aliquot of TBME. The process was repeated. Crudes were subsequently dried in a desiccator over silica gel after which the solids were dissolved in a water methanol mixture and purified by RP-LCMS. Pure fractions were combined and lyophilized.

2.2 NODA-dLVT

After peptide synthesis, the resin was retrieved, washed and swelled in DMF for 1 h. Solvent was removed and a solution of 2% v/v hydrazine monohydrate in DMF was added. The mixture was agitated for 1 h, the deprotection mixture removed, and the resin washed. This process was repeated and resin bound peptide was used directly for conjugation. For this, HATU (44 mg, 115 μmol) in DMF (200 μL) was added dropwise to a mixture of NODA-MPAA (61 mg, 120 μmol),[2] and DIPEA (21 μL, 120 μmol) in DMF (400 μL). The mixture was agitated for 5 min then added to a DMF slurry of DIPEA (21 μL, 120 μmol) and resin bound dLVT (40 μmol)

with the free lysine residue. The mixture was agitated for a further 2.5 h, after which the solution was removed by suction and washed. Resin was subsequently dried in a desiccator overnight then deprotected and cleaved using a mixture of TFA/DTT/H_2O/TIS (91 : 3 : 3 : 3) for 3 h. Crude mixture was precipitated, centrifuged and dried in a dessicator, after which time the solid was dissolved and purified by RP-LCMS. Following lypolization a white solid was afforded (16 mg, 29%). Cyclisation was achieved using phosphate buffer (0.01 M, pH 7.5), at room temperature with 15% v/v DMSO (at a peptide concentration of 1 mg/ml) for 48 h. The peptide was purified by preparative RP-LCMS, affording NODA-dLVT as a white solid after lyophilisation (77%).

2.3 AlF-NODA-dLVT

The synthesis of AlF-NODA-dLVT followed a similar protocol to one reported elsewhere.[3] Briefly, NODA-dLVT (2 mg, 1.45 μmol) was dissolved in 2 mM sodium acetate buffer (110 μl, pH 4.4) and ethanol (55 μL), and treated with $AlF_3.3H_2O$ (1.1 mg, 7.25 μmol). The pH was adjusted to 4.8 and the reaction mixture was refluxed for 90 min. The mixture was cooled, the crude diluted five-fold and purified by preparative RP-LCMS to yield cold tracer as a white solid after lyophilization (1.8 mg, 87%).

2.4 Reduced AlF-NODA-dLVT

AlF-NODA-dLVT (0.5 mg, 0.35 μmol) was dissolved in ammonium bicarbonate (0.1 M, pH 8) at a concentration of 1 mg/ml, and dithiothreitol (0.54 mg, 3.5 μmol) was added. The mixture was left to stand at room temperature for 3 h, then purified by LCMS affording reduced peptide after lyophilisation (0.4 mg, 80%).

2.5 Synthesis of OT and peptide analogues

Synthesis of reduced open chain peptides followed the general procedure. Once completed, peptides were cleaved using the usual deprotection mixture and work up. Peptides were

dissolved in a water methanol mixture and purified by RP-LCMS. Oxidation, when required, was performed in air with 0.1 M ammonium hydrogen carbonate buffer (peptide concentration of 0.1 mg/ml) for up to 3 days, after which the solution was concentrated, then lyophilised to yield pure peptides.

3 NODA-dLVT labelling with $Al^{18}F$

Care was taken to ensure reactants were prepared in metal free conditions. A stock solution of NODA-dLVT in water was made. Aliquots (72 nmol) were removed and placed in eppendorfs for lyophilization. For the preparation of buffer solutions, any pH adjustment made used metal free glacial acetic acid.

3.1 Preparation of $Na^{18}F$

In a typical procedure a QMA cartridge was washed with 0.5 M sodium acetate buffer (10 ml, pH 8.4), followed by deionized water (10 ml), as described previously.[4] [^{18}F]fluoride solution (6-10 mL; 1 GBq) was loaded onto the cartridge, washed with water (2 ml) and $Na^{18}F$ was eluted with 20% saline solution into 100 µL fractions. The fraction containing the highest activity (790 MBq) was subsequently used for radiolabelling.

3.2 Synthesis of [^{18}F]AlF-NODA-dLVT

To eluted $Na^{18}F$, 2 mM $AlCl_3$ (10 µL, 20 nmol) in 0.1 M sodium acetate buffer, pH 4.1, was added. The solution was added to a mixture of NODA-dLVT (72 nmol) in 0.5 M NaOAc buffer (10 µL; pH 4.0). The reaction mixture was incubated at 100 ^{o}C for 15 min and purified by manual RP-HPLC, affording isolated tracer.

Radiolabelling time: The length of reaction was studied using a molar ratio of 0.6:1 aluminium chloride to peptide with 20% saline (*n*=3). ^{18}F yields were not improved through extending the reaction time past 15 min: at 5 min, ^{18}F yield was 40 ± 8% and at 15 min the labelling efficiency

was 84 ± 2%. After 30 min ^{18}F yields remained at 84 ± 3%.

4 Biological studies

4.1 Tissue Preparation for rat brain autoradiography

Male Wistar rats (250 g; *n*=3) were stunned then decapitated. Brains were rapidly removed, incubated in 20% sucrose solution for 2 h, and immediately frozen (-80°C). Tissues were stored at -80°C until required. Rat brains were sectioned in the sagittal plane (20 µm thickness; according to the atlas of Paxinos and Watson, 1998). Tissues were cut using a cryostat microtome (CM3050S, Leica, UK), and thaw-mounted onto glass microscope slides. Slides were stored at -80 °C until use.

4.2 Autoradiography Procedure

Tissue was thawed to room temperature, dried then washed with ice cold buffer (50 mM Tris, pH 7.4). Sections were air dried and incubated for 1 h at room temperature with [^{18}F]AlF-NODA-dLVT (3 nM) in buffer (50 mM Tris pH 7.4, 10 mM $MgCl_2$). For nonspecific binding, OT (10 µM) was co-incubated with [^{18}F]AlF-NODA-dLVT (3 nM) over the duration of the experiment. After incubation, slides were washed with ice-cold buffer (2 min × 2), followed by a final wash in ice-cold distilled water (2 min). Slides were dried in a cool airstream then exposed to ^{18}F sensitive phosphor screens (Amersham, UK) with [^{18}F]AlF-NODA-dLVT standards in X-ray cassettes overnight. Phosphor screens were imaged using a Typhoon 8600 scanner (Molecular Dynamics) and images were analysed with OptiQuant 5.0 (Perkin Elmer) and FIJI. Values were converted to relative binding percentages using calibrated [^{18}F]AlF-NODA-dLVT standards. Binding potential (BP) is defined as the binding of tracer in brain region/total activity incubated as determined by the standard. Specific binding in each region is calculated by comparing the total and non-specific BP by the equation: ((total BP – non-specific BP)/total BP) × 100.

4.3 In vivo procedures

All animal experiments were performed in accordance to the Animals (Scientific Procedures) Act 1986 and were approved by the King's college London ethical review committee. The work was performed under PPL 70/8230 (Reza Rezavi). Adult female Wistar rats (220 ± 4.5 g) were group housed at 21 ± 1 °C in a 12 h light:dark cycle with ad libitum access to standard rat chow and water. [^{18}F]AlF-NODA-dLVT was formulated in 0.9% saline to contain no more than 5% MeOH. Female Wistar rats received a dose of [^{18}F]AlF-NODA-dLVT by tail vein injection (up to 650 µL in volume), or *via* nasal administration using a syringe with a plastic cannula attached to one nostril (25 µL per nare).

4.4 i.n. administration

A 0.3 mL insulin syringe, containing a 29-gauge needle, was fitted with a PE10 cannula (15 mm). The cannula was fitted so that it covered approximately 3 mm of the needle tip. Radioligand was added to the syringe and the activity measured before and after administration to calculate the injected dose (ID). Anesthesia was induced using a mixture of 5% isoflurane and oxygen (Animalcare, UK) at a flow rate of 1 L/min and maintained at 2.5% isoflurane. Once unresponsive, the rat was held in an almost vertical position and radioactivity was administered through one nare at the back of the nasal cavity.

4.5 Biodistribution

Rats were sacrificed by removal of the heart under isoflurane anaesthesia at 10 min following tail vein injection or 30 min following *i.n.* administration. Major organs were excised, washed with saline, weighed and counted in a gamma counter (LKB Wallac 1282), together with standards of the injection mixture. Organ uptake was calculated as percentage injected dose per gram [%ID/g] of tissue mass. Data are reported as mean ± SD.

4.6 Statistical analysis

Statistical analysis was carried out using the unpaired Student's t test algorithm on GraphPad Prism software. Findings were deemed significant if p values were less than 0.05.

Name	Sequence	MW (Da)	ES (m/z)	Rt
OT_{oc}	C Y I Q N C P L G	1006.4	1007.9 (m+1)	9.6
dOT_{oc}	dC Y I Q N C P L G	993.4	994.7 (m+1)	11.8
dOT_{c}	dC Y I Q N C P L G	991.4	992.6 (m+1)	11.7
$dLVT_{oc}$	dC Y I Q N C P K G	1008.5	1009.7 (m+1)	9.0
$dLVT_{c}$	dC Y I Q N C P K G	1006.4	1007.5 (m+1)	7.0
AlF-NODA-$dLVT_{oc}$	dC Y I Q N C P **K*** G	1427.6	1428.7 (m+1)	10.0
AlF-NODA-$dLVT_{c}$	dC Y I Q N C P **K*** G	1425.6	1424.9 (m-1)	10.2

Supplementary Table 1: Primary sequences and characterisation data for peptides screened in OT and VP receptor binding assays. Analysis of all peptides were conducted on a Waters LC-MS system. Key: dC = deaminated cysteine; K* = ε amino group conjugated to NODA, as in Figure 1; $_{oc}$ = open chain; $_{c}$ = cyclised. Disulphide bonds occur between residues 1 and 6 when cyclised (c), or are disulphide bridged with the corresponding xylene shown in subscript.

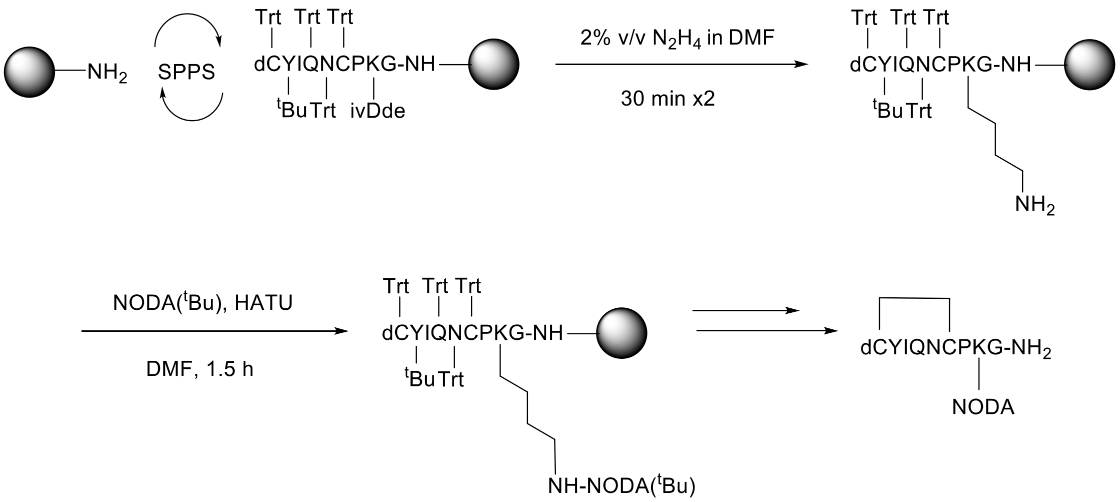

Supplementary Scheme 1: Synthetic route for production of OTR PET tracer NODA-dLVT by automated SPPS. Following incorporation of deaminated cysteine in the final position, lysine is selectively deprotected on resin allowing conjugation of NODA. Deprotection, cleavage and cyclisation affords the tracer precursor.

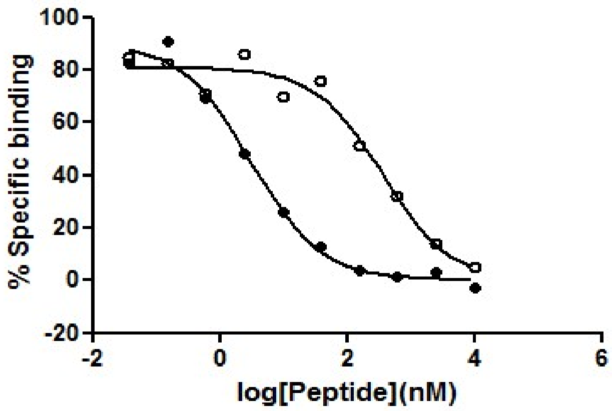

Supplementary Figure 1: Dose-response curve of AlF-NODA-dLVT binding to human OTR (black points) or human V1a receptors (white points), showing selectivity towards the OTR.

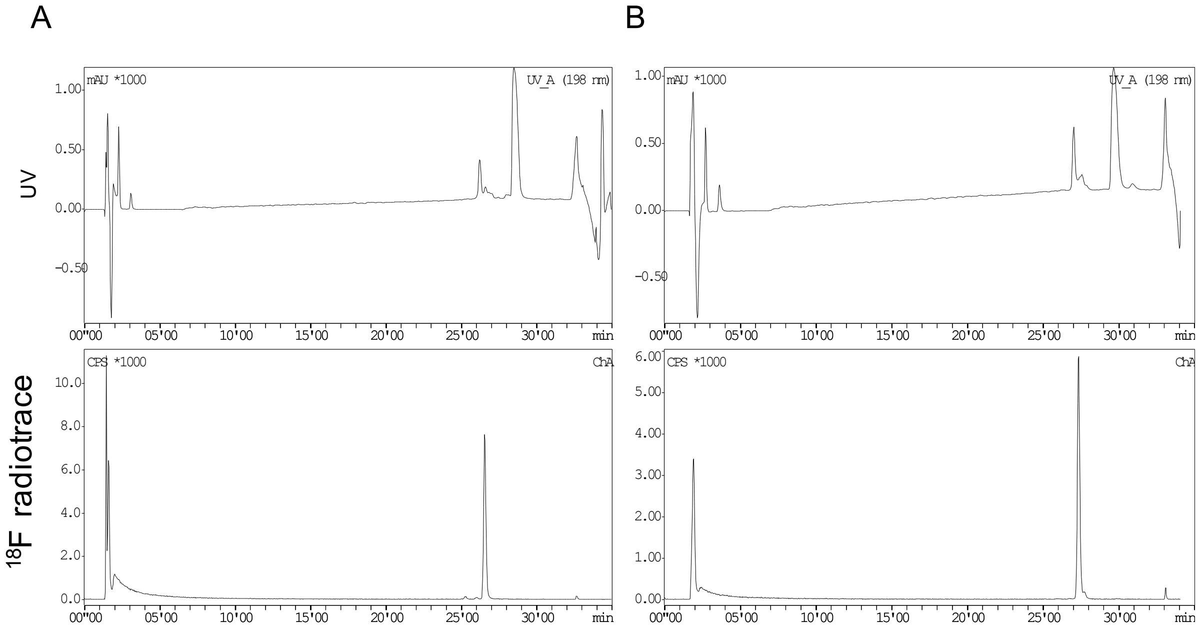

Supplementary Figure 2: Typical chromatograms for [^{18}F]AlF labelling of NODA-dLVT when 0.9% (A) or 20 % saline (B) is used at 105 °C for 15 min. Unbound Al^{18}F is eluted in the void volume, while labelled peptide elutes at 27 min and unlabelled precursor is at 29 min.

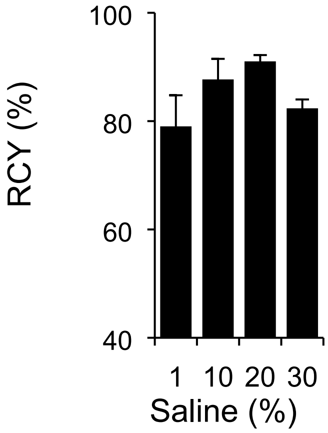

Supplementary Figure 3: RCC for labelling of NODA-GAFG (72 nmol) with [^{18}F]AlF under different saline concentrations at 100 °C for 15 min with $AlCl_3$ (20 nmol).

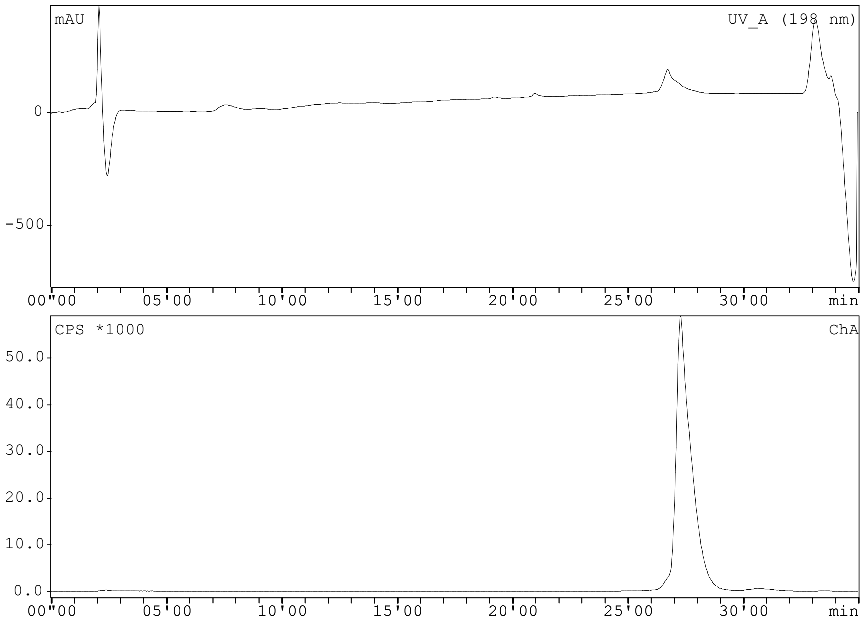

Supplementary Figure 4: HPLC trace of purified [^{18}F]AlF- NODA-dLVT. UV is shown on top whilst ^{18}F radiotrace is on the bottom.

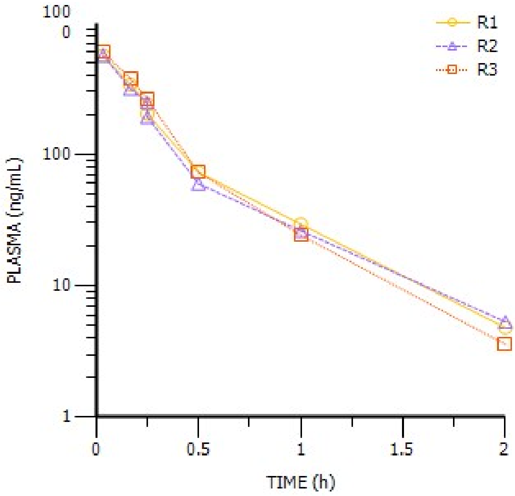

Supplementary Figure 5: Pharmacokinetic data acquired from three individual rats showing plasma clearance of AlF-NODA-dLVT over time. Rats received a dose of AlF-NODA-dLVT by tail vein injection (0.08 mg/kg), and blood plasma samples were taken at the specified time point for quantitative analysis by mass spectrometry. Each rat (n=3) was sampled up to 9 times over the course of 24 h.

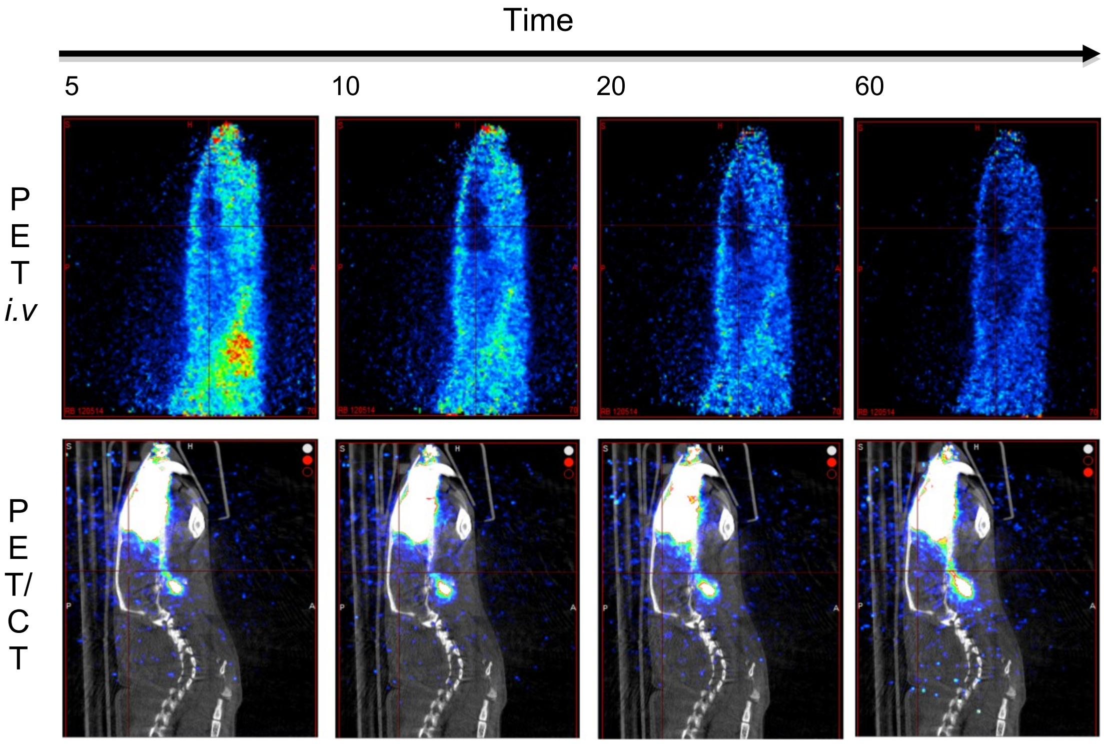

Supplementary Figure 6: Transaxial nanoPET scan showing distribution over time of [^{18}F]AlF-NODA-dLVT. Tracer was administered either by *i.v.* bolus (top) or intranasally (bottom) in rat.

References

1. Ratni, H., Rogers-Evans, M., Bissantz, C., Grundschober, C., Moreau, J. L., Schuler, F., Fischer, H., Alvarez Sanchez, R., Schnider, P. (2015) Discovery of highly selective brain-penetrant vasopressin 1a antagonists for the potential treatment of autism via a chemogenomic and scaffold hopping approach. *J Med Chem, 58*, 2275-89.
2. Sharma, K. S., Durand, G., Giusti, F., Olivier, B., Fabiano, A.-S., Bazzacco, P., Dahmane, T., Ebel, C., Popot, J.-L., Pucci, B. (2008) Glucose-Based Amphiphilic Telomers Designed to Keep Membrane Proteins Soluble in Aqueous Solutions: Synthesis and Physicochemical Characterization. *Langmuir, 24*, 13581-13590.
3. D'Souza, C. A., McBride, W. J., Sharkey, R. M., Todaro, L. J., Goldenberg, D. M. (2011) High-Yielding Aqueous 18F-Labeling of Peptides via Al18F Chelation. *Bioconjugate Chem, 22*, 1793-1803.
4. McBride, W. J., D'Souza, C. A., Sharkey, R. M., Karacay, H., Rossi, E. A., Chang, C.-H., Goldenberg, D. M. (2010) Improved ^{18}F Labeling of Peptides with a Fluoride-Aluminum-Chelate Complex. *Bioconjugate Chem*, *21*, 1331-1340.
